# Gender Differences in Post-Stroke Spasticity Patients Treated with OnabotulinumtoxinA: Insights from the BOTOX Economic Spasticity Trial (BEST) †

**DOI:** 10.3390/toxins18020064

**Published:** 2026-01-26

**Authors:** Monica Verduzco-Gutierrez, Reema Kaloti, Akinpelumi A. Beckley, Adil Syed Hussain, Sima A. Desai, Kimberly Becker Ifantides, Natasha L. Romanoski

**Affiliations:** 1 Long School of Medicine, University of Texas at San Antonio, San Antonio, TX 78229, USA; 2 AbbVie Inc., North Chicago, IL 60064, USA; kim.becker@abbvie.com; 3 Department of Rehabilitation and Regenerative Medicine, Columbia University Medical Center, New York, NY 10032, USA; aab2236@cumc.columbia.edu; 4 Rancho Los Amigos National Rehabilitation Center, Downey, CA 90242, USA; ahussain@atsu.edu; 5 Atrium Health Carolinas Rehabilitation, Charlotte, NC 28203, USA; sima.desai@advocatehealth.org; 6 Department of Physical Medicine and Rehabilitation, Penn State College of Medicine, Hershey, PA 17033, USA; nromanoski@pennstatehealth.psu.edu

**Keywords:** onabotulinumtoxinA, botulinum neurotoxin A, stroke, spasticity, gender differences, post-stroke spasticity, pain, rehabilitation, health disparities

## Abstract

Limited data are available on gender differences in patients with post-stroke spasticity (PSS) treated with onabotulinumtoxinA (onabotA). This subgroup analysis of data from the BOTOX^®^ Economic Spasticity Trial (BEST) focused on onabotA-treated patients stratified by gender. BEST was a double-blind, placebo-controlled, randomized study with an open-label extension that allowed for up to five treatments. It evaluated the effectiveness of onabotA + Standard of Care (SC) vs. placebo + SC for the treatment of PSS. At baseline, out of 139 patients treated with onabotA, females (*n* = 54) had a slightly higher body mass index (BMI) compared to males (*n* = 85) (28.3 vs. 26.9 kg/m^2^), and a greater percentage of females (40.7%) took analgesic medications compared to males (31.8%). Scores from baseline assessments for pain, spasticity severity, and stroke recovery were comparable between groups. Despite these similarities at baseline, females received an average lower dose (range, 337–365 U) of onabotA compared to males (range, 343–421 U) across treatment sessions. Females also had lower changes from baseline compared to males on pain, spasticity severity, stroke recovery, and functional goal achievement assessments across most onabotA treatment sessions. There is a need for further investigation into treatment approaches to optimize outcomes for both males and females with PSS.

## 1. Introduction

Stroke is among the leading causes of death and disability in the world today, with an estimated 101 million prevalent cases reported in 2019, resulting in approximately 6.5 million deaths and 143 million disability-adjusted life years [1]. Women accounted for 56% of these prevalent cases, and research has shown that they have a higher lifetime risk of and poorer outcomes for stroke [2,3,4,5,6]. The potential biological causes for sex- and gender-based discrepancies in stroke incidence and outcome are difficult to disentangle from confounding factors such as age and socioeconomic status, but they may be attributable to higher rates of common risk factors associated with stroke, such as hypertension and diabetes, as well as sex-specific risk factors such as pregnancy and menopause [4,5].

Spasticity is a common complication of central nervous system damage, including stroke, which is characterized by intermittent or sustained involuntary muscle activation resulting in stiffness or tightness of muscles [7,8,9,10,11]. Post-stroke spasticity (PSS) can be disabling and is frequently associated with abnormal limb posture, joint contracture, pain, and reductions in quality of life [7,8,9,10]. OnabotulinumtoxinA (OnabotA) is a botulinum toxin approved for the treatment of both upper- and lower-limb spasticity, as supported by randomized controlled clinical trials that demonstrated notable reductions in disability, pain, and muscle tone [12,13,14,15,16]. Patients and clinicians have reported high satisfaction with the effectiveness of onabotA for spasticity [17]. Patients were more likely to participate in therapy and exercise, experienced reductions in disability, and reported lower spasticity-related pain after onabotA treatment [17]. There is limited data available on gender differences in patients with spasticity and their outcomes, with or without onabotA treatment.

The BOTOX^®^ Economic Spasticity Trial (BEST) was a randomized, double-blind, placebo-controlled study designed to evaluate the impact of incorporating onabotA treatment into standard of care for patients with PSS [18]. The study, which concluded in 2010, found that the addition of onabotA to standard of care resulted in significant increases in physical goal attainment, higher levels of active function, and greater reductions in pain compared to placebo [19,20]. The primary objective of this post hoc analysis of BEST was to identify differences in post-stroke functional goal achievement, pain, spasticity severity, and stroke recovery between genders (male and female) at baseline and after treatments with onabotA. Our findings demonstrate that female patients with PSS were given lower doses of onabotA and had lower changes from baseline across key efficacy endpoints across the trial period.

## 2. Results

### 2.1. Baseline Demographics and Patient Clinical Characteristics

The Intent-to-Treat population (ITT) included 273 patients, 139 of whom were randomized to onabotA (Table 1). Of these, 61.2% (85/139) were male, which was comparable to the total ITT population (58.6% [160/273]). Baseline characteristics were comparable between male and female subgroups, with some exceptions: males were older (63.0 vs. 57.9 years), had a higher average body weight (82.0 vs. 74.3 kg), lower body mass index (BMI) (26.9 vs. 28.3 kg/m^2^), and were less likely to take analgesics (31.8 vs. 40.7%). Both groups were primarily Caucasian and had similar baseline scores for Numerical Pain Rating Scale (NPRS), REsistance to Passive movement Scale-26 (REPAS-26), and Stroke Impact Scale-16 (SIS-16). In the female cohort, 53.7% (29/54) of patients received injections in both upper and lower limbs at baseline, compared to 44.7% (38/85) in males; no patients received baseline injections on both sides (right and left) of the body.

### 2.2. OnabotA Dosage Across Treatment Visits

**Figure 1:**
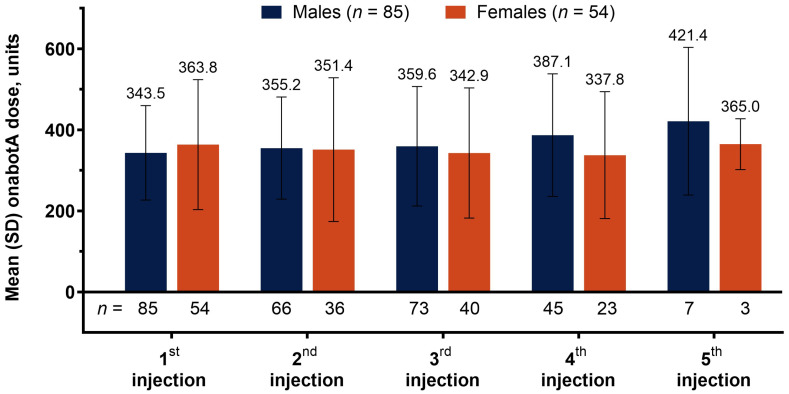
Total onabotA dose administered across treatments. Abbreviations: *n*, number of patients in each subgroup treated with onabotA (top) and across treatment visits (bottom); onabotA, onabotulinumtoxinA; SD, standard deviation.

Males were given a lower mean (SD) dose of onabotA for the 1st (baseline) injection of 343.5 (116.7) units (U) compared to 363.8 (160.4) U given to females (Figure 1). Dosage for males consistently increased with each subsequent visit, to 355.2 (125.9), 359.6 (147.5), and 387.1 (151.3) U at the 2nd, 3rd, and 4th injections, respectively. Simultaneously, female patients primarily experienced a reduction in onabotA dosage, to 351.4 (177.4), 342.9 (160.6), and 337.8 (156.6) U at the 2nd, 3rd, and 4th injections, respectively. Only 10 patients received a 5th injection, with male patients receiving 421.4 (182.3) U of onabotA and female patients receiving 365.0 (62.7) U at this visit.

### 2.3. Achievement of Principal Active Functional Goal as Measured by the Goal Attainment Scale Across Treatments

**Figure 2:**
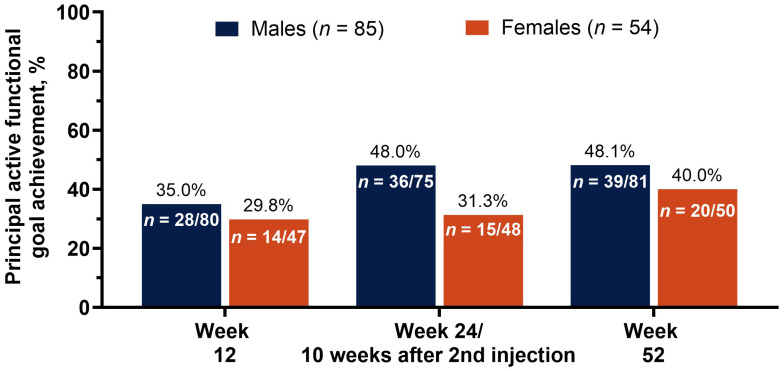
Proportion of patients who achieved their primary active functional goal at each treatment. Goal attainment was assessed at pre-specified postbaseline visits: week 12, week 24, or 10 weeks after 2nd injection, and week 52. Abbreviations: *n*, number of patients in each subgroup treated with onabotA (top) and number of patients in each subgroup across treatment visits (bottom).

The male subgroup had a higher rate of principal active functional goal attainment across all post-baseline visits (Figure 2). At the first assessment (week 12), 35.0% of males achieved their goal vs. 29.8% of females. The difference was highest at the conclusion of the double-blind treatment period (week 24 or 10 weeks after the optional 2nd injection), with 48.0% of males vs. 31.3% of females achieving their goal at this visit. At the final assessment (week 52), the rate of goal attainment for males was maintained at 48.1%, while the rate for females increased to 40.0%.

### 2.4. Reduction in Pain as Measured by NPRS Score Across Treatments

**Figure 3:**
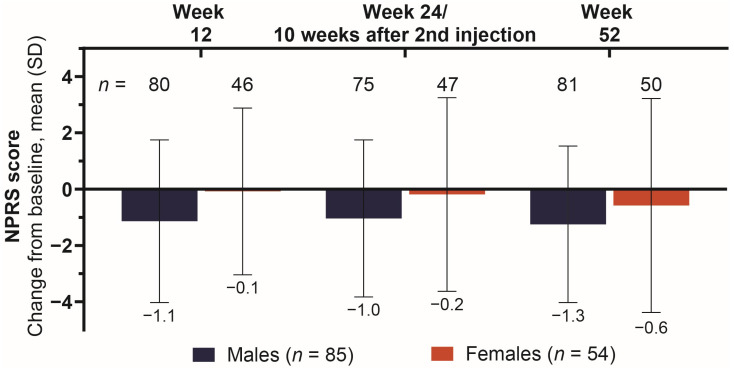
Mean change from baseline in pain score across onabotA treatments. Abbreviations: *n*, number of patients in each subgroup across treatment visits (top) and number of patients treated with onabotA (bottom); NPRS, Numeric Pain Rating Scale; onabotA, onabotulinumtoxinA; SD, standard deviation.

The male subgroup had a greater reduction in pain (NPRS score) compared to females across all post-baseline visits (Figure 3). Change from baseline was consistent in the male subgroup, with means (SD) of −1.1 (2.9), −1.0 (2.8), and −1.3 (2.8) at the 1st, 2nd, and 3rd assessments, respectively. The improvement in pain score for females was comparatively minimal, especially during the double-blind treatment phase, where the mean (SD) for this subgroup was −0.1 (3.0) and −0.2 (3.4). Mean (SD) change from baseline was −0.6 (3.8) among females at the conclusion of the study.

### 2.5. Changes in Severity of Spasticity Measured by REPAS-26 Score Across Treatments

**Figure 4:**
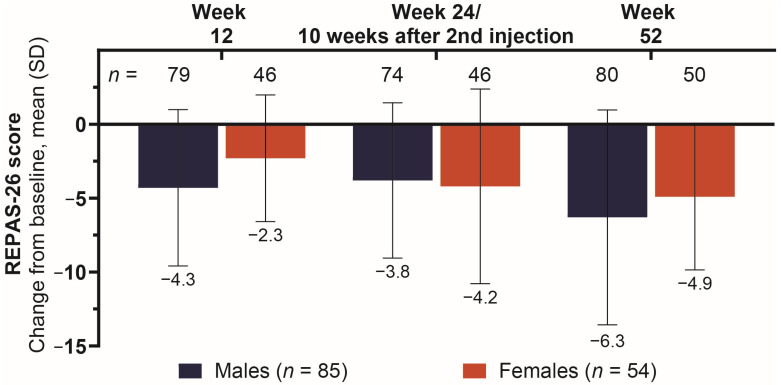
Mean change from baseline in resistance to passive movement. Abbreviations: *n*, number of patients in each subgroup across treatment visits (top) and number of patients treated with onabotA (bottom); REPAS-26, Resistance to PAssive movement Scale-26; onabotA, onabotulinumtoxinA; SD, standard deviation.

The male subgroup had a greater mean (SD) reduction in REPAS-26 score at weeks 12 [−4.3 (5.3) vs. −2.3 (4.3)] and 52 [−6.3 (7.3) vs. −4.9 (5.0)] compared to females (Figure 4). At the second visit assessment (week 24 or 10 weeks after first injection), improvement in REPAS-26 scores was similar between the groups, with males reporting a mean (SD) change from baseline of −3.8 (5.3) vs. females reporting a change of −4.2 (6.6).

### 2.6. Changes in Stroke Severity as Assessed by Stroke-Related Physical Function on the SIS-16 Score Across Treatments

**Figure 5:**
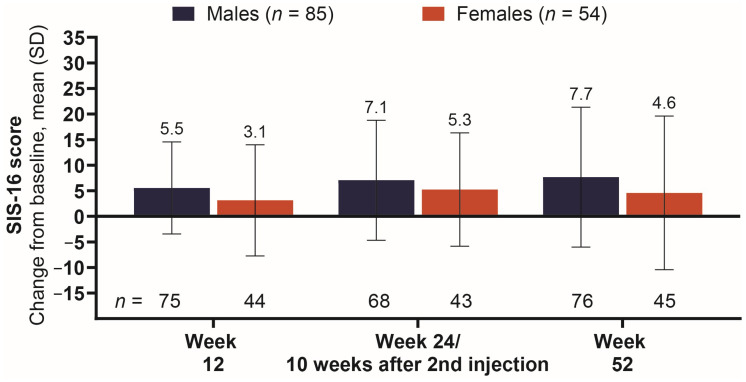
Mean change from baseline in post-stroke recovery of physical function. Abbreviations: *n*, number of patients in each subgroup treated with onabotA (top) and number of patients in each subgroup across treatment visits (bottom); SIS-16, Stroke Impact Scale-16; onabotA, onabotulinumtoxinA; SD, standard deviation.

The male subgroup had a greater improvement in stroke-related physical function (SIS-16 score) compared to females across all post-baseline visits (Figure 5). Change from baseline consistently increased in the male subgroup, with a mean (SD) of 5.5 (9.0), 7.1 (11.7), and 7.7 (13.7) at the 1st, 2nd, and 3rd assessments, respectively. At the first assessment, mean (SD) for SIS-16 was 3.1 (10.9) among females, increasing to 5.3 (11.1) by the second assessment and then decreasing to 4.6 (15.0) by study conclusion.

### 2.7. Safety Overview

Over the duration of the study (double-blind and open-label phase), males were less likely to experience a treatment-emergent adverse event (TEAE) than females (63.5% vs. 75.9%) (Table 2). Of these, 14 male patients (16.5%) reported 19 serious TEAEs, while 16 female patients (29.6%) reported 28 serious TEAEs. Only one of these serious TEAEs was determined to be related to the administration of onabotA: a male patient reported worsening of residual hand function of the spastic limb, possibly because of onabotA causing local muscle weakness; the patient did not withdraw from the study. The most common TEAEs were fall, nasopharyngitis, and musculoskeletal pain. The greatest disparity in TEAEs was for musculoskeletal pain, with nine (10.6%) of the male subgroup reporting this event, vs. only one (1.9%) in the female subgroup.

## 3. Discussion

Data from BEST has shown that the addition of onabotA to standard of care is an effective treatment for PSS, with patients experiencing improvements in pain, goal attainment, spasticity severity, and stroke recovery compared to placebo [16,18,19,20]. Recent research has suggested that women may be more prone to stroke and less responsive to therapies for stroke, but little is known about the gender disparity in PSS, which is common among stroke patients. To our knowledge, this post hoc analysis of onabotA-treated patients from BEST is the first study to compare outcomes for male and female patients treated for PSS. Future studies are necessary to confirm these findings, as this exploratory analysis is limited by a small patient population and may not fully represent the diversity of patients affected by the condition.

Of the 139 patients randomized to onabotA at baseline, 54 (38.8%) reported their gender as female, and this cohort was 5.1 years younger, on average. This is in contrast with data from real-world evidence studies, which have shown that female stroke patients are about 5 years older than males [4,5,21]. The female cohort had higher BMIs, despite males being heavier at baseline. Obesity is more prevalent and serves as a stronger risk factor for stroke in females compared to males [4,5], yet BMI does not account for physiological differences in body composition related to fat mass, muscle mass, or bone density. Body composition may also vary widely between males and females due to lifestyle, hormonal, or biological factors such as a history of pregnancy or post-menopausal state. Spasticity outcomes and the influence of onabotA on muscle properties related to body composition are yet to be determined. At baseline, the two cohorts had similar scores for pain, spasticity severity, and stroke recovery, although a higher proportion of females in our study took analgesic medication, which may have brought down the average pain score in the female cohort at baseline and throughout the study period. Additionally, differences in pain between males and females throughout the study could further be attributed to differences in hormonal influences, neurological pathways, and pain sensitivity. Across the study, onabotA was effective and well-tolerated in both males and females.

The BEST study protocol states that a minimum onabotA dose was pre-specified according to consensus from the lead investigators’ clinical practice, and that each administered dose was determined based on individual investigators’ experience, and depended on the size, number, and location of the muscles being treated, the severity of spasticity, and the presence of local muscle weakness. Despite the similarities in baseline characteristics, female patients were treated with an additional 20.3 U of onabotA compared to males in the initial dose, on average. Notably, female patients were more likely (53.7%) than male patients (44.7%) to receive injections in both upper and lower limbs at baseline.

The onabotA dose increased with each subsequent treatment for male patients (343.5, 355.2, 359.6, 387.1, 421.4 from 1st to 5th injection, respectively), while the dosage mostly decreased for females patients (363.8, 351.4, 342.9, 337.8, 421.4 from 1st to 5th injection, respectively), with the 5th injection representing a very small number of patients and the only dosage increase in the female cohort. As a result, the disparity in onabotA dosage increased with each injection, from −20.3 U (males − females) at baseline to 3.8 U, 16.7 U, 49.3 U, and 56.4 U at injections 2, 3, 4, and 5, respectively. The dose increase that occurred with each subsequent treatment in the male cohort reflects common practice, as clinicians aim to optimize muscle/dose selection over a few treatment cycles; however, the dose reduction seen in females for the 2nd, 3rd, and 4th treatments, despite starting with a higher dose, may reflect biases and disparities in practice patterns. Of note, females reported a higher rate of TEAEs, which may have resulted in the treating physician reducing the dose to mitigate these adverse events. When considering gender differences specific to spasticity, the temporal pattern of onabotA treatments should be considered, along with individualized treatment plans.

Twelve weeks after the baseline injection, male patients had better outcomes than female patients across all assessments, including principal active functional goal achievement, pain score, spasticity severity, and stroke recovery. The relative difference in onabotA dosage went from 20.3 U in favor of female patients (1st injection) to 3.8 U in favor of male patients (2nd injection), despite (or perhaps because of) the stronger response to treatment in the male cohort. The favorable outcomes in the male cohort at 12 weeks, despite lower starting dosages, may be due to factors beyond onabotA dosage, for which gender-specific variables should be explored.

At the 24-week (or 10 weeks after 2nd injection) assessment, principal active functional goal attainment increased substantially among male patients, from 35.0% to 48.0%, while goal achievement was largely unchanged among females (29.8% to 31.3%). The change from baseline in pain score was similar for both cohorts between week 12 and week 24 or 10 weeks after 2nd injection (−1.1 to −1.0 vs. −0.1 to −0.2 for males vs. females, respectively), whereas the change from baseline in stroke recovery (as measured by SIS-16 score) improved in both cohorts, from 5.5 to 7.1 in males and 3.1 to 5.3 in females, with male patients still experiencing a higher change from baseline in stroke recovery score. The only assessment in which female patients had better outcomes than male patients at any point in this study was for spasticity severity (REPAS-26) at week 24 or 10 weeks after 2nd injection, where the change from baseline in REPAS-26 for males went from −4.3 to −3.8 vs. females who experienced an improvement from −2.3 to −4.2. Further consideration for physiological factors may identify why males still had a larger improvement in goal achievement and stroke recovery, despite the larger reduction in spasticity severity for females.

At the 52-week assessment, female patients showed improvements from the previous assessment in principal functional goal attainment (31.3% to 40.0%), pain score (−0.2 to −0.6), and spasticity severity (−4.2 to −4.9), but not in stroke recovery (5.3 to 4.6). Male patients at week 52 had similar rates of goal attainment from the previous assessment (48.0% to 48.1%) and saw improvements in pain score (−1.0 to −1.3), spasticity severity (−3.8 to −6.3), and stroke recovery (7.1 to 7.7).

More research is needed to determine how factors related to gender differences affect spasticity and how management may need to be tailored based on gender. By evaluating the effectiveness of the intervention and adjusting the treatment plan based on individual responses, providers can enhance therapeutic efficacy and achieve better outcomes for all patients.

Given the age of this dataset and the recent call to action from the AAPM&R consensus guidance on spasticity and management [22] in improving treatment equity, the authors recommend a commitment to collaboration between healthcare institutions, insurers, drug manufacturers, professional societies, and advocacy groups to openly engage in highlighting the evidence gaps in spasticity management, particularly as stated in this manuscript between females and males. Elucidating the potential need for tailored treatments by gender and utilizing such evidence for clinical decision-making may improve equity and increase access to botulinum toxin therapy in spasticity management.

### Limitations

This analysis is exploratory and utilizes descriptive statistics; hence, these results should be interpreted with caution. BEST was not originally designed to test hypotheses specific to gender differences, as evidenced by the relatively small proportion of females among the onabotA cohort and the age disparity between males and females. Furthermore, while multicenter designs are a strength in prospective randomized clinical trials, they may increase heterogeneity when applied to a limited post hoc dataset. Gender-related differences observed in this analysis may be confounded by other factors not accounted for in the original study design, including differences in age, pre-stroke disability, and location of spasticity (upper or lower limb) between gender cohorts. Future studies aimed at this topic should have closer to equal representation of male and female patients and ask for investigators to provide clinical reasoning for changes in dosage.

Given the timing (completed in 2010) and location (Canada, Germany, Sweden, and the United Kingdom) of the BEST study, dosing and other treatment parameters may have been influenced by limited published data available at the time to support optimal muscle selection and dosing, as well as the absence of dosing guidance from current onabotA labels and medical guidelines addressing best practices among female and male post-stroke spasticity patients. Dosing recommendations, maximum total dose limits, goal-oriented treatment strategies, and awareness of gender-related treatment equity have evolved considerably in the last 15 years. Consequently, the dosing patterns and longitudinal trends observed in this analysis may reflect historical clinical practice rather than mechanisms directly applicable to the contemporary management of spasticity. Another limitation of this analysis is the categorization of gender as binary (male and female), which does not account for non-binary and other gender identities.

Analysis of efficacy in patients who experienced either an increase or a decrease in dose over time was limited due to the small sample size of the subgroups. Furthermore, the apparent dosing differences between the male and female cohorts are clinically minimal compared to the total administered dose and may fall within the expected variability in routine spasticity management. In the absence of statistical tests or dose–response analyses, it is difficult to determine whether these differences are clinically meaningful. Interpretation is further limited by the observational nature of the analysis, potential confounding by baseline disease severity, treatment goals, injector practice patterns, and unmeasured factors that may influence dosing decisions over time. Hence, it cannot be concluded whether the greater improvement in male patients compared to female patients was due to an increase in dose over subsequent visits, other confounding factors, or random variation.

## 4. Conclusions

This subgroup analysis of BEST compared treatment dosage and outcomes between male and female participants who were treated with onabotA during the double-blind and open-label phases of this 52-week trial. The safety profile for onabotA treatment for both groups was consistent with the prescribing information; no new safety signals were identified. Despite similar baseline levels of spasticity, females received lower doses of onabotA than males, on average. Females also had lower changes from baseline compared to males on pain, spasticity severity, and stroke recovery assessments across most onabotA treatments. This exploratory analysis was limited by a small sample size that precluded the use of inferential statistics; hence, future studies are necessary to confirm and expand on these results. Nonetheless, our findings highlight the importance of assessing the patient’s response to treatment and tailoring the therapy accordingly to ensure optimal outcomes.

## 5. Materials and Methods

### 5.1. Study Design and Patient Population

BEST (NCT-00549783) was a multicenter, double-blind, prospective, placebo-controlled, randomized Phase 3b study that was conducted in Canada, Germany, Sweden, and the United Kingdom from 2007 to 2010. The study evaluated the effectiveness of onabotA + Standard of Care (SC) vs. placebo + SC for the treatment of adult patients with PSS. The study design and protocol have been previously published [18,19,20]. The study population included patients aged 18–85 with a diagnosis of focal upper and/or lower limb spasticity following a stroke (due to a primary cerebral hemorrhage/infarction) that occurred ≥3 months before the screening visit. Patients with fixed contracture as a result of spasticity or causes of spasticity other than stroke were excluded from this study.

**Figure 6:**
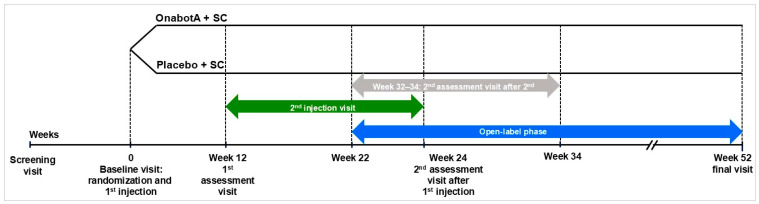
BEST study design. Abbreviations: onabotA, onabotulinumtoxinA; SC, standard of care.

Demographic data, including gender and race/ethnicity, were self-reported by patients at the screening visit. Gender was collected as male or female. Patients were randomized 1:1 to onabotA + SC or placebo + SC at the baseline visit (week 0), followed by a first assessment visit (week 12), an optional second injection (12–24 weeks), a second assessment (24 weeks, or 10 weeks after second injection if given), and a final assessment at 52 weeks (Figure 6). The study period consisted of 22–34 weeks of double-blind treatment, followed by an open-label phase. During the open-label phase, all patients were eligible to receive onabotA injections, with a minimum inter-injection interval of 12 weeks. Patients were administered up to 800 units of study treatment at each treatment session.

### 5.2. Outcome Measures and Assessments

The primary objective of BEST was to assess the number of patients who achieved their principal functional goal at the conclusion of the double-blind treatment period (week 24 or 10 weeks after the second injection), as determined by their physician. Secondary endpoints described in this subanalysis included the number of patients achieving their principal functional goal at weeks 12 and 52, changes in NPRS, REPAS-26, and SIS-16 scores from baseline to each study visit, and documentation of TEAEs during the study.

A principal active functional goal was determined at baseline for each patient, with input from the patient and physician together. These were defined as improvement in active function of an upper/lower limb to achieve a personal goal, e.g., dressing, feeding, washing, climbing stairs, or walking. Physicians underwent training prior to the study to ensure that goals were realistic, achievable, and meaningful to the patient [23]. Goal attainment scaling was measured using a 6-point scale, from −3 (function is worse than baseline) to 0 (goal achievement) to +2 (improvements much greater than expected); goal attainment was defined as a score of 0, +1, or +2.

Pain was assessed using the 11-point NPRS, ranging from 0 (no pain) to 10 (pain as bad as can be imagined). A reduction of 30% in the NPRS represents a clinically important difference in patients with chronic pain [24]. REPAS-26 is a validated scale for assessing resistance to passive movement and was used to demonstrate changes in the severity of spasticity [25]. It consists of 13 passive arm and leg motions on either side of the body across different joints, each of which are rated according to the Modified Ashworth Scale, from 0 (no increase in muscle tone) to 4 (limb rigid in flexion or extension). SIS-16 is a stroke-specific measure that summarizes aspects of stroke recovery across 16 domains pertaining to physical function [26].

Patients were monitored for signs and symptoms of AEs throughout the study. Serious AEs were defined as those causing death or those that were life-threatening, required hospitalization, or resulted in persistent or significant morbidity.

### 5.3. Statistical Analyses

These analyses were not prespecified in the protocol and no inferential statistical analyses were conducted. Results for patient demographics, clinical characteristics, and safety are reported as the number and percentage of patients. All efficacy and safety data are presented for the ITT population, which included all enrolled patients who were randomized and received treatment at baseline. All other variables are provided using descriptive statistics, e.g., mean (SD). No imputation was performed for missing data. Patient gender was collected as either male or female.

## Figures and Tables

**Table 1:** Baseline demographics and patient clinical characteristics.

Parameter at Baseline	Males (*n* = 85)	Females (*n* = 54)
Age (years)		
Mean (SD)	63.0 (10.4)	57.9 (13.4)
Age category (years), *n* (%)		
<65	40 (47.1)	33 (61.1)
≥65	45 (52.9)	21 (38.9)
Race, *n* (%)		
Caucasian	84 (98.8)	52 (96.3)
Black/African American	1 (1.2)	2 (3.7)
Asian/South East Asian	0	2 (3.7)
BMI, kg/m^2^		
*n*	85	53 ^a^
Mean (SD)	26.9 (3.8)	28.3 (5.1)
Weight, kg		
*n*	85	53 ^a^
Mean (SD)	82.0 (11.0)	74.3 (13.9)
Location of baseline injection, *n* (%)		
Upper limb only	22 (25.9)	14 (25.9)
Lower limb only	25 (29.4)	11 (20.4)
Upper and lower limbs	38 (44.7)	29 (53.7)
Concomitant medications, *n* (%)		
Analgesics	27 (31.8)	22 (40.7)
Oral anti-spasmodic	20 (23.5)	13 (24.1)
NPRS (pain) score		
*n*	85	53 ^a^
Mean (SD)	3.4 (2.9)	3.4 (3.3)
REPAS-26 score		
*n*	84 ^a^	53 ^a^
Mean (SD)	22.1 (9.2)	18.9 (8.9)
SIS-16 score		
*n*	82 ^a^	52 ^a^
Mean (SD)	59.8 (19.2)	58.5 (20.7)

^a^ Missing data for some patients. Abbreviations: BMI, body mass index; *n*, number of patients in each subgroup treated with onabotA at baseline; NPRS, Numerical Pain Rating Scale, onabotA, onabotulinumtoxinA; REPAS-26, REsistance to PAssive movement Scale-26 (spasticity severity); SIS-16, Stroke Impact Scale-16 (stroke recovery, or stroke impact on physical function); SD, standard deviation.

**Table 2:** Overview of treatment-emergent adverse events.

Parameter at Baseline	Males (*n* = 85)	Females (*n* = 54)
TEAEs		
*n*	164	131
Patients with ≥1, *n* (%)	54 (63.5)	41 (75.9)
Serious TEAEs		
*n*	19	28
Patients with ≥1, *n* (%)	14 (16.5)	16 (29.6)
Serious, drug-related TEAEs		
*n*	1	0
Patients with ≥1, *n* (%)	1 (1.2)	0 (0)
TEAEs leading to withdrawal		
*n*	0	0
Patients with ≥1, *n* (%)	0 (0)	0 (0)
Patients with ≥1 specific TEAEs, *n* (%) ^a^		
Fall	8 (9.4)	7 (13.0)
Nasopharyngitis	6 (7.1)	5 (9.3)
Musculoskeletal pain	9 (10.6)	1 (1.9)
Urinary tract infection	4 (4.7)	4 (7.4)
Pain in extremity	5 (5.9)	2 (3.7)
Muscular weakness	3 (3.5)	4 (7.4)

^a^ Specific TEAEs reported by ≥5% of patients in either study population are reported. Abbreviations: TEAE, treatment-emergent adverse event; *n*, number of events reported or number of patients; %, percentage of patients reporting ≥1 event out of the intention-to-treat population.

## Data Availability

AbbVie is committed to responsible data sharing regarding the clinical trials we sponsor. This includes access to anonymized, individual, and trial-level data (analysis data sets), as well as other information (e.g., protocols, clinical study reports, or analysis plans), as long as the trials are not part of an ongoing or planned regulatory submission. This includes requests for clinical trial data for unlicensed products and indications. These clinical trial data can be requested by any qualified researchers who engage in rigorous, independent, scientific research, and will be provided following review and approval of a research proposal, Statistical Analysis Plan (SAP), and execution of a Data Sharing Agreement (DSA). Data requests can be submitted at any time after approval in the US and Europe and after acceptance of this manuscript for publication. The data will be accessible for 12 months, with possible extensions considered. For more information on the process or to submit a request, visit the following link: https://vivli.org/ourmember/abbvie/ (accessed on 26 June 2025) then select “Home”.

## Abbreviations

The following abbreviations are used in this manuscript:

PSS	Post-stroke spasticity
OnabotA	OnabotulinumtoxinA
BEST	BOTOX^®^ Economic Spasticity Trial
SC	Standard of Care
NPRS	Numeric Pain Rating Scale
REPAS-26	REsistance to PAssive movement Scale-26
SIS-16	Stroke Impact Scale-16

## References

[1] GBD 2019 Stroke Collaborators (2021). Global, Regional, and National Burden of Stroke and Its Risk Factors, 1990–2019: A Systematic Analysis for the Global Burden of Disease Study 2019. Lancet Neurol..

[2] Feigin V.L., Brainin M., Norrving B., Martins S., Sacco R.L., Hacke W., Fisher M., Pandian J., Lindsay P. (2021). World Stroke Organization (WSO): Global Stroke Fact Sheet 2022. Int. J. Stroke.

[3] Seshadri S., Beiser A., Kelly-Hayes M., Kase C.S., Au R., Kannel W.B., Wolf P.A. (2006). The Lifetime Risk of Stroke. Stroke.

[4] Yoon C.W., Bushnell C.D. (2023). Stroke in Women: A Review Focused on Epidemiology, Risk Factors, and Outcomes. J. Stroke.

[5] Ospel J., Singh N., Ganesh A., Goyal M. (2023). Sex and Gender Differences in Stroke and Their Practical Implications in Acute Care. J. Stroke.

[6] Xu M., Vallejo A.A., Calvete C.C., Rudd A., Wolfe C., O’Connell M.D.L., Douiri A. (2022). Stroke Outcomes in Women: A Population-Based Cohort Study. Stroke.

[7] Kuo C.-L., Hu G.-C. (2018). Post-Stroke Spasticity: A Review of Epidemiology, Pathophysiology, and Treatments. Int. J. Gerontol..

[8] Zorowitz R.D., Gillard P.J., Brainin M. (2013). Poststroke Spasticity. Neurology.

[9] Sommerfeld D.K., Gripenstedt U., Welmer A.-K. (2012). Spasticity After Stroke. Am. J. Phys. Med. Rehabil..

[10] Pandyan A., Gregoric M., Barnes M., Wood D., Wijck F.V., Burridge J., Hermens H., Johnson G. (2005). Spasticity: Clinical Perceptions, Neurological Realities and Meaningful Measurement. Disabil. Rehabil..

[11] Wissel J., Manack A., Brainin M. (2013). Toward an Epidemiology of Poststroke Spasticity. Neurology.

[12] Botox. Prescribing Information. AbbVie Inc.. https://www.rxabbvie.com/pdf/botox_pi.pdf.

[13] Shaw L.C., Price C.I.M., Wijck F.M.J., van Shackley P., Steen N., Barnes M.P., Ford G.A., Graham L.A., Rodgers H., Investigators B. (2011). Botulinum Toxin for the Upper Limb After Stroke (BoTULS) Trial. Stroke.

[14] Wissel J., Ward A.B., Erztgaard P., Bensmail D., Hecht M.J., Lejeune T.M., Schnider P., Altavista M.C., Cavazza S., Deltombe T. (2009). European Consensus Table on the Use of Botulinum Toxin Type A in Adult Spasticity. J. Rehabil. Med..

[15] Rosales R.L., Chua-Yap A.S. (2008). Evidence-Based Systematic Review on the Efficacy and Safety of Botulinum Toxin-A Therapy in Post-Stroke Spasticity. J. Neural Transm..

[16] Wissel J., Ri S. (2022). Assessment, Goal Setting, and Botulinum Neurotoxin a Therapy in the Management of Post-Stroke Spastic Movement Disorder: Updated Perspectives on Best Practice. Expert Rev. Neurother..

[17] Francisco G.E., Jost W.H., Bavikatte G., Bandari D.S., Tang S.F.T., Munin M.C., Largent J., Adams A.M., Zuzek A., Esquenazi A. (2020). Individualized OnabotulinumtoxinA Treatment for Upper Limb Spasticity Resulted in High Clinician- and Patient-Reported Satisfaction: Long-Term Observational Results from the ASPIRE Study. PMR.

[18] Borg J., Ward A.B., Wissel J., Kulkarni J., Sakel M., Ertzgaard P., Åkerlund P., Reuter I., Herrmann C., Satkunam L. (2011). Rationale and Design of a Multicentre, Double-Blind, Prospective, Randomized, European and Canadian Study: Evaluating Patient Outcomes and Costs of Managing Adults with Post-Stroke Focal Spasticity. J. Rehabil. Med..

[19] Ward A.B., Wissel J., Borg J., Ertzgaard P., Herrmann C., Kulkarni J., Lindgren K., Reuter I., Sakel M., Säterö P. (2014). Functional Goal Achievement in Post-Stroke Spasticity Patients: The BOTOX^®^ Economic Spasticity Trial (BEST). J. Rehabil. Med..

[20] Wissel J., Ganapathy V., Ward A.B., Borg J., Ertzgaard P., Herrmann C., Haggstrom A., Sakel M., Ma J., Dimitrova R. (2016). OnabotulinumtoxinA Improves Pain in Patients with Post-Stroke Spasticity: Findings From a Randomized, Double-Blind, Placebo-Controlled Trial. J. Pain Symptom Manag..

[21] Appelros P., Stegmayr B., Terént A. (2009). Sex Differences in Stroke Epidemiology. Stroke.

[22] Verduzco-Gutierrez M., Raghavan P., Pruente J., Moon D., List C.M., Hornyak J.E., Gul F., Deshpande S., Biffl S., Lawati Z.A. (2024). AAPM&R Consensus Guidance on Spasticity Assessment and Management. PM&R.

[23] Ertzgaard P., Ward A., Wissel J., Borg J. (2011). Practical Considerations for Goal Attainment Scaling during Rehabilitation Following Acquired Brain Injury. J. Rehabil. Med..

[24] Farrar J.T., Young J.P., LaMoreaux L., Werth J.L., Poole R.M. (2001). Clinical Importance of Changes in Chronic Pain Intensity Measured on an 11-Point Numerical Pain Rating Scale. Pain.

[25] Platz T., Vuadens P., Eickhof C., Arnold P., Kaick S.V., Heise K. (2008). REPAS, a Summary Rating Scale for Resistance to Passive Movement: Item Selection, Reliability and Validity. Disabil. Rehabil..

[26] Duncan P.W., Lai S.M., Bode R.K., Perera S., DeRosa J. (2003). Stroke Impact Scale-16. Neurology.

